# Changes in peripheral oxytocin and vasopressin during a silent month-long Insight meditation retreat

**DOI:** 10.3389/fendo.2024.1345527

**Published:** 2024-05-28

**Authors:** Quinn A. Conklin, Anthony P. Zanesco, Brandon G. King, Elissa S. Epel, Clifford D. Saron

**Affiliations:** ^1^ Center for Health and Community, University of California, San Francisco, San Francisco, CA, United States; ^2^ Center for Mind and Brain, University of California, Davis, Davis, CA, United States; ^3^ Department of Psychology, University of Miami, Miami, FL, United States; ^4^ Department of Psychiatry, University of California, San Francisco, San Francisco, CA, United States; ^5^ The MIND Institute, University of California, Davis, Davis, CA, United States

**Keywords:** meditation, oxytocin, vasopressin, prosociality, social interaction, attachment style

## Abstract

**Background:**

Given its putative roles in mediating prosocial behavior, attachment bonds, and stress physiology, oxytocin modulation has been hypothesized to be a biological correlate of the salubrious effects of meditation practice. Here we investigated the effects of a month-long silent meditation retreat on changes in oxytocin, and the related hormone and vasopressin, in relation to psychosocial changes in attachment style, anxiety, personality measures, and feelings of social connectedness with fellow meditators.

**Methods:**

Plasma oxytocin and vasopressin and self-report questionnaires were measured in retreat participants (*n* = 28) at the beginning of, and 3 weeks into, a residential meditation retreat. Control participants (*n* = 34), who were similar in age, gender, and meditation experience, were also assessed across a 3-week interval. Linear mixed effects models were used to assess outcomes.

**Results:**

The retreat group showed a small but significant decrease in oxytocin compared to controls who showed no change. In the retreat group, higher openness to experience at Time 1 predicted greater reductions in oxytocin during the retreat, and lower oxytocin at Time 2 was related to stronger feelings of personal connection with fellow meditators. The changes in oxytocin were not related to attachment style or anxiety. Vasopressin decreased over time across both groups, suggesting no specific effect of retreat.

**Conclusion:**

These preliminary findings suggest that meditation training in the context of a silent residential retreat may reduce circulating levels of oxytocin. We interpret this finding from multiple theoretical perspectives, discussing key measurement limitations and proposing future study designs that may help to differentiate the effects of different meditation practices and contexts on oxytocin signaling.

## 1 Introduction

Rapid technological advances and increasing global connectivity have changed the way we experience age-old human challenges, including psychosocial stress, loneliness, and interpersonal conflict. We now have a near constant ability to access news and communicate with people from around the world, which has dramatically increased the rate of information we are processing and our exposure to potentially stressful stimuli. These challenges underscore the need for skillful approaches to better regulate our attention and to cultivate mental habits that reduce our own and others’ suffering in the world. Attention regulation, stress reduction, and prosocial behavior are among the purported outcomes of meditation training that have gained considerable interest in health psychology as avenues for improving individual and interpersonal well-being. At the same time, given its putative roles in mediating prosocial behavior and stress buffering, changes in the oxytocin system have been hypothesized to be one of the biological mechanisms underlying the psychosocial benefits of meditation training—particularly forms of compassion and loving-kindness meditations that are believed to promote prosocial orientations toward others (1–3). Here we investigate changes in plasma oxytocin, and the related hormone vasopressin, during a month-long meditation retreat to better understand the psychobiological consequences of intensive meditation training.

Oxytocin and vasopressin are neuropeptide hormones implicated in the regulation of social cognition, behavior, and communication, as well as several homeostatic and reproductive functions. Both neuropeptides are primarily synthesized in the hypothalamus from which they are secreted within the brain and released into the bloodstream from the posterior pituitary (4). Oxytocin is also synthesized in several peripheral sites including the heart, uterus, and placenta (5). The pleiotropic functions of these hormones are achieved through a combination of distinct neuronal populations governing different functions (4, 6, 7) and regional distribution patterns of the oxytocin receptor, OXTR, and the vasopressin receptors, V1aR, V1bR, and V2 (4). Because of their structural similarities, they are also able to bind to each other’s receptors (8).

Oxytocin was first recognized for its role in stimulating uterine contractions and milk let down after birth (Dale, 1906 reviewed in 9). Subsequent research has underscored its role in social attachment, including maternal caregiving (10), infant-parentbonding (11), and pair bonding in adults (12, 13). Like oxytocin, arginine vasopressin is involved in social recognition, communication, aggression, maternal care, pair bonding, and avoidance and anxiety-like behavior in response to stressful situations (14). However, vasopressin—also known as antidiuretic hormone—is best understood for its roles in water homeostasis and blood pressure regulation (15), and has been the topic of less research in human behavior because vasopressin manipulation can have potentially negative side effects, including increased blood-pressure and disrupted kidney function (16). Interestingly, both human and animal studies suggest that there can be sexually dimorphic patterns in the effects of oxytocin and vasopressin on specific social behaviors—with one or the other hormone playing a stronger role in particular behaviors depending on the sex of the individual (4, 14, 17–19).

Early theories focused oxytocin’s role in prosocial behavior and affiliative motivation (20)—with research suggesting that oxytocin is involved in a number of social emotions and behaviors including trust (21, 22), generosity (23, 24), and empathy (24–26). In a recent study, Akinrinade and colleagues (27) demonstrated that oxytocin was necessary and sufficient to elicit fear contagion, behavioral distress, and consoling behavior in zebrafish—suggesting that oxytocin’s role in empathic processes is evolutionarily conserved in social species (27). Studies have also shown that positive attachment is associated with higher plasma oxytocin in women (28) and that intranasal oxytocin enhances attachment security in men (29).

Along these lines, oxytocin is thought to be a primary mediator of the stress-buffering effects of social support (30, 31). Evidence across species suggests that oxytocin is released in response to several physiological and psychological stress paradigms and that the oxytocin system modulates hypothalamic-pituitary-adrenal (HPA), autonomic, immune, and behavioral responses to stress (6). In humans, oxytocin has been shown to increase in response to the Trier Social Stress Test (32–34). These oxytocin increases are positively correlated with increases in cortisol—though the associations are small (35), and the evidence that oxytocin attenuates cortisol responses is weak and seemingly context specific (36).

Given oxytocin’s role in social emotions, behavior, and stress physiology, Mascaro et al. (1) hypothesized that the oxytocin system may be involved in mediating the effects of meditation on prosocial emotions and behavior—suggesting that oxytocin’s widespread neuromodulatory effects may provide a parsimonious explanation for the multitude of effects observed in response to kindness-based meditation (1). Luberto et al. (2) further hypothesized that meditation may promote relaxation-responses and prosocial outcomes by stimulating the oxytocin system and mimicking the physiology of secure attachment (2).

Despite these compelling hypotheses, very few empirical studies have investigated the relationship between oxytocin and meditation practice. Two initial studies found positive correlations between oxytocin and measures of spirituality (37, 38), suggesting that meditation, which is often conceived of as a spiritual practice, may relate to oxytocin expression. In line with these findings, Van Cappellen and colleagues (39) found that intranasally administered oxytocin increased self-reported spirituality in men and greater levels of positive emotions after an introductory meditation session. Additionally, the observed increases in spirituality were mediated by variants of the CD38 gene, which is involved in the release of oxytocin from hypothalamic neurons (39).

Only two published studies have examined circulating oxytocin levels in relation to meditation interventions. One study found increases in salivary oxytocin alongside increases in self-reported empathy and empathic accuracy in students undergoing a 2-month mindfulness and compassion-based intervention (40). In this same cohort, Bellosta-Batalla and colleagues (41) reported increased salivary oxytocin alongside reductions in anxiety and negative emotions after a single laboratory-based mindfulness meditation session, as compared to an emotion recognition exercise (41). In another long-term training study, Hoehne and colleagues (42) found that after 3 months of compassion-based meditation training, participants showed lower overall plasma oxytocin levels during the Trier Social Stress Test and higher oxytocin levels during a single session of loving-kindness meditation (42). As with the broader social behavior literature, there is less research on the relationships between meditation and vasopressin than oxytocin, though one early study reported increases in vasopressin after a transcendental meditation session (43).

The goal of the present study was to assess the effects of month-long Insight meditation retreat on changes in plasma oxytocin and vasopressin. To date, most empirical studies of meditation and mindfulness have been based on single-session mindfulness inductions, relatively brief training programs lasting a few days, or 6- to 8-week programs designed for beginning or novice practitioners [e.g., mindfulness-based stress reduction, MBSR; (44)]. The total duration of meditation practice in such programs is relatively low compared to more traditional approaches to contemplative training, which often involve several hours of meditation practice each day for periods ranging from several days to many months. By contrast, meditation retreats afford individuals the opportunity to practice meditation to a greater extent than is typically possible given the demands of daily life (45). Retreats are usually designed as residential programs involving structured periods of formal practice with guidance from experienced teachers and social support from similarly motivated individuals. To reduce the distractions of daily life, retreats are often held in secluded natural environments in facilities designed for such a purpose. Practitioners may also engage in noble silence—a practice that entails temporarily refraining from speaking, communicating, or engaging in eye contact during the retreat in order to facilitate focus on one’s inner experience.

By design, meditation retreats alter practitioners’ behavioral patterns (e.g., hours of formal meditation each day), physical surroundings (e.g., serene natural environments) and social context (e.g., maintaining silence and refraining from eye-contact). Due to their immersive nature, retreats may elicit changes in psychobiological processes that are less malleable to non-intensive interventions (45). The meditation tradition studied here also emphasizes ‘nonattachment’, which involves relating to one’s own experiences and to other people with less rigidity/reactivity and with increased flexibility/composure (46), and an ethical framework that prizes loving-kindness, compassion, empathetic joy, and equanimity (47). This style of training has been shown to promote improvements in attention regulation (48, 49), shifts in empathic responses to suffering (50), profound psychological insights (51), and improvements in adaptive psychological functioning, including attachment security (48).

Given prior work demonstrating increases in oxytocin associated with meditation interventions (40, 42), we expected increases in oxytocin during the the month-long retreats studied here. In line with the hypothesis that oxytocin might mediate meditation-related improvements in attachment style and prosociality (2), we further predicted that oxytocin increases would be associated with improvements in attachment security and greater feelings of social connectedness. To test the anxiolytic implications of oxytocin regulation, we also assessed changes in oxytocin in relation to previously-reported reductions in anxiety in this same retreat group (52)—predicting that increases in oxytocin may be related to decreases in anxiety. Finally, because our prior work has indicated that personality factors can predict biological outcomes in the context of intensive retreats, we assessed changes in oxytocin in relation to the Big Five personality dimensions (52, 53). Given the paucity of research on meditation and vasopressin, we did not have strong predictions regarding the changes we might observe in vasopressin.

## 2 Materials and methods

The data presented in this study are secondary outcomes from a larger investigation of intensive meditation training approved by the University of California, Davis Institutional Review Board (ClinicalTrials.gov #NCT03056105). The purpose of the broader study was to evaluate the effects of an ecologically valid month-long residential retreat program on psychobiological outcomes. Additional details regarding the meditation training, retreat environment, and participant recruitment and demographics can be found in our prior reports (51, 52, 54, 55).

### 2.1 Participants

We recruited two self-selected groups of participants. Retreat participants (*n* = 28; 14 female, 13 male, 1 nonbinary; mean age 51.9 years, range 27–69) were recruited from two month-long meditation retreats held annually at Spirit Rock Meditation Center in Woodacre, CA. To participate in these retreats, applicants were required to have previously attended at least two retreats of 7 days or longer. Control participants (*n* = 34; 23 female, 11 male; mean age 49.3 years, range 25–68) were recruited from the larger Spirit Rock meditation community through flyers and presentations at weekly classes. The control participants were selected to be similar to the retreat group in age, gender, and prior meditation retreat experience (see 52), and were encouraged to maintain their usual meditation practice routines for the duration of the study. Because the primary focus of the study was to investigate the effects of meditation on telomere length, applicants were excluded if they reported medical conditions that might influence telomere biology or immune cell distributions, including cancers, autoimmune diseases, immunodeficiency disorders, or other conditions involving chronic inflammation such as hepatitis. Participants were not excluded for use of antidepressant or anxiolytic medication.

All participants gave informed consent before taking part in the study and were compensated at a rate of approximately $20 per hour for their participation in the assessments. Retreat participants were assessed in February and March of 2013 and controls were assessed in small groups from May 2013 through February of 2014. Two retreat participants withdrew from the retreat after the first assessment for reasons unrelated to the study and did not complete the Time 2 blood draw or questionnaires. Three control participants withdrew from the study after the initial blood draw due to scheduling conflicts or overall time commitment, and one control participant completed the Time 2 questionnaires but not the blood draw. Four additional control participants failed to complete some portion of the questionnaires: two did not complete the questionnaires at either time point, one did not complete the Time 1 questionnaires, and one did not complete the Time 2 questionnaires.

### 2.2 Procedure

#### 2.2.1 Retreat intervention

The meditation interventions were two month-long, silent, residential retreats. Participants received instruction in Insight meditation—a form of vipassana meditation rooted in the Theravadan Buddhist tradition. Core practices taught during these retreats included mindfulness of breathing and directing one’s focus to present moment-experience (56). Participants also received instruction in practices intended to cultivate aspirational qualities known as the four immeasurables or Brahma Viharas: loving-kindness, compassion, empathetic joy, and equanimity (57, 58). The retreat schedule included ~10 hours of formal meditation each day practice (alternating between sitting and walking practice in 30 to 45-minute intervals), meals, rest, and an hour of work meditation (e.g., kitchen or housekeeping duties) (52). To facilitate quietude and inner focus, participants maintained noble silence—refraining from eye contact and verbal communication, except for regular meetings with teachers.

#### 2.2.2 Blood collection and self-report assessments

Blood samples were collected by professional phlebotomists via antecubital venipuncture. Retreat participants were assessed onsite at Spirit Rock Meditation Center and control participants were assessed at the Anubhuti Retreat Center in Novato, CA—a similarly isolated and quiet center near Spirit Rock. Retreat participants gave blood between 5:00 and 6:00 AM on the morning following their first full day of retreat^1^ and again 3 weeks later. Control participants were also assessed at the beginning and end of a 3-week interval, during which they maintained their typical daily routines. Control participants gave blood between 9:00 and 10:00 AM after completing a 40-minute meditation session^2^. Both groups were asked to fast for at least 8 hours prior to their blood draw. Participants’ weight was measured immediately before each blood collection, and their height was recorded at the second assessment. BMI was calculated as BMI = [Weight (lbs) * 703]/Height (in)^2^. After each blood draw, participants were given a packet of questionnaires and asked to complete and return them to the retreat manager’s office (retreats group) or by mail (control group) within 36 hours.

At each assessment, a total of 45.5 ml of blood was collected from each participant. One tube containing 6ml was collected for plasma isolation in a BD vacutainer coated with K2 EDTA as an anticoagulant (BD 367863) and placed directly on ice. Samples were transported to a laboratory every 20 minutes to ensure that processing began within 45 minutes of collection. At the lab, plasma tubes were inverted 10 times and then centrifuged for 15 minutes at 2750 rpm at 4°C. The plasma-separator tubes were allowed to rest for 10 minutes before 200 µL aliquots of plasma were pipetted into 8 cryovials and transferred to dry ice. These aliquots were then transported on dry ice to the University of California, Davis, where they were stored in a -80°C freezer (ThermoFisher Forma 88400) until September of 2014 when they were delivered on dry ice to Dr. Karen Bales’ laboratory at the University of California, Davis for assay.

### 2.3 Measures

#### 2.3.1 Oxytocin and vasopressin assays

Samples were kept frozen until they were assayed. Oxytocin was assayed using the Oxytocin ELISA kit from Enzo Life Sciences (Farmingdale, NY, cat # ADI-900–153A, lot # 03261411A) per the manufacturer’s protocol, with the exception that the extraction step was not conducted^3^. According to the product manual, the sensitivity of this assay was 15.0pg/mL and the cross-reactivity for arg^8^-vasopressin was 7.5%. Plasma samples were diluted 1:8 with assay buffer and compared to standard concentrations of 1,000 pg/mL-15.6pg/mL. Intra assay CVs for oxytocin were < 9%. The intra-class correlation (ICC) for oxytocin duplicates was 0.82, CI 95% [0.734, 0.895]^4^. No plasma was collected for 1 retreat participant at Time 1.

Arg^8^-Vasopressin was also measured with an ELISA kit from Enzo Life Sciences (cat # ADI-901–017, lot # 08191407) per the manufacturer’s protocol, with the exception that 3 samples had too little material to be run in duplicate and were only measured once. According to the product manual, the sensitivity of this assay was 3.39 pg/mL and the cross-reactivity with oxytocin was <0.001%. Plasma samples were diluted 1:2 and compared to a standard curve of 1,000pg/mL-4.1pg/mL for Arg^8^-Vasopressin. Intra assay CVs for vasopressin were < 11%. The ICC for vasopressin duplicates was 0.74, 95% CI [0.57, 0.86]^4^. The vasopressin assay resulted in 18 samples that were undetectable (8 retreat samples—including both samples for 4 participants; and 10 control samples—including both samples for 4 participants, the Time 1 sample for 1 participant, and the Time 2 sample for another participant). Another 6 samples fell below the lower limit of the standard curve; these values were retained in the analyses presented in the results.

#### 2.3.2 Self-report measures

Attachment style was measured using the 12-item Experiences in Close Relationships-12 (ECR-12) questionnaire (63). The ECR-12 consists of two subscales: attachment anxiety (6 items) and attachment avoidance (6 items). Example items include: “I worry about being rejected or abandoned” (anxiety) and “I don’t feel comfortable opening up to others” (avoidance). Participants were asked to indicate their level of agreement with each item using a 7-point Likert scale ranging from (1) strongly disagree to (7) strongly agree. Possible scores for the two subscales range from 1 to 7, with higher scores indicating higher attachment insecurity. One control participant did not complete this scale at the Time 1 assessment, and another control participant did not complete it at Time 2.

Anxiety was assessed with the 10 trait items from the State-Trait Anxiety Inventory [STAI-T; (64)]. Example items include: “I feel nervous and restless” and “I am a steady person” (reverse scored). Participants were asked to indicate how often they felt a particular way over the past month on a 4-point Likert scale ranging from (1) almost never to (4) almost always. Items were summed to calculate scores with a possible range of 20 to 80. Higher scores indicate higher anxiety.

Personality dimensions were measured with the 44-item Big Five Personality Inventory (65). The Big Five measures extraversion (8 items), agreeableness (9 items), conscientiousness (9 items), neuroticism (8 items), and openness to experience (10 items). Example items include “I see myself as someone who is curious about many different things” (openness) or “…is outgoing, sociable” (extroversion). Participants were asked to indicate their level of agreement with each item using a 7-point Likert scale ranging from (1) strongly disagree to (7) strongly agree. Subscale items were averaged to calculate subscale scores with a possible range of 1 to 7. Higher scores indicate a greater degree of a given personality trait. One retreat participant did not complete this scale at the Time 1 assessment.

Lifestyle dimensions including health responsibility (9 items), physical activity (8 items), nutrition (9 items), spiritual growth (9 items), interpersonal relations (9 items), and stress management (8 items) were assessed with the 52-item Lifestyle Profile II (66). Participants indicated the frequency with which they engaged in listed behaviors using a 4-point Likert scale ranging from (1) never to (4) routinely. Items were averaged to calculate subscale scores with a possible range of 1 to 4. Higher scores indicate greater engagement with a particular lifestyle factor.

Meditation practice and feelings of connectedness with fellow meditators were assessed at Time 2. Participants were asked to estimate how many hours per week they had practiced during the study period. They were also asked to report the degree to which they felt personally connected to other meditators in their community on a 7-point scale ranging from (1) strongly disagree to (7) strongly agree.

### 2.4 Data processing and statistical analyses

All analyses were conducted in R version 4.2.2 and R studio version 2023.6.1.524. Oxytocin and vasopressin data were transformed using the natural log (ln) to improve normality and reduce heteroscedasticity, as indicated by Box-Cox tests (using the ‘boxcox’ function), Shapiro-Wilk’s tests of normality (using ‘shapiro_test’ from the *rstatix* package), and Levene’s tests of homogeneity (using ‘levene_test’ from the *rstatix* package). These variables were then evaluated for outliers using the median absolute deviation (MAD) approach (67) applied to the total data set (i.e., with groups and time points combined) with a cutoff of +/- 3 * MAD. Two oxytocin observations (1 retreat participant at Time 1, and 1 control participant at Time 2) and one vasopressin observation (1 retreat participant at Time 1) were identified as outliers and winsorized to +/- 3 * MAD using the ‘winsorize’ function (from the *DescTools* package). We also evaluated whether these cases were associated with extreme change scores (i.e., Time 2 - Time 1) to identify outliers that might unduly influence our change-for-change correlation analyses. One participant was identified as having an extreme oxytocin change score and was subsequently removed from all analyses. Alternative analyses using the winsorized values for this participant did not change the overall pattern of results.

We assessed the effects of retreat on oxytocin, vasopressin, and attachment using linear mixed effects models with the ‘lme’ function from the *nlme* package. Because MLMs can accommodate missing data, participants who withdrew from the study or were unable to complete all assessments were included in analyses. We fit separate models for oxytocin, vasopressin, and attachment anxiety and avoidance to assess the main effects of Group, Time, and the Group x Time interaction, with random intercepts for participants. The *emmeans* package was used to calculate orthogonal pairwise comparisons and correct for multiple comparisons using the Tukey-Kramer method for each model. Breusch-Pagan’s test for heteroscedasticity (‘bptest’ from the *lmtest* package) indicated that the variance in oxytocin values differed between the retreat and control groups, so unique variances were specified for each group in the model. Given the sexually dimorphic effects sometimes observed in oxytocin, we also examined separate models for men and women.

We used the ‘hedges_g’ function from the *easystats* package to calculate the effect sizes for the between group differences at Time 1 and Time 2 (reported in **Table 1**) and the within-group changes between assessments (reported in **Table 2**) using the raw data.

**Table 1:** Summary statistics by time and group.

Variable	Time 1								Time 2							
	Retreat			Control			Group Difference		Retreat			Control			Group Difference	
	*N*	*Mean*	*SD*	*N*	*Mean*	*SD*	*g*	*95% CI*	*N*	*Mean*	*SD*	*N*	*Mean*	*SD*	*g*	*95% CI*
Oxytocin Ln (pg/ml)	26	7.72	0.25	34	7.79	0.39	0.22	[-0.29, 0.72]	26	7.63	0.26	30	7.82	0.33	0.64*	[0.11, 1.17]
Vasopressin Ln (pg/ml)	23	3.56	1.06	27	3.33	1.05	-0.22	[-0.77, 0.33]	20	3.28	0.93	23	3.10	0.89	-0.02	[-0.78, 0.40]
Attachment Anxiety (1-7)	28	3.38	1.27	29	3.54	1.73	0.10	[-0.41, 0.62]	25	3.26	1.30	27	3.35	1.50	0.06	[-0.48, 0.60]
Attachment Avoidance (1-7)	28	2.74	1.24	29	2.68	1.11	-0.05	[-0.56, 0.46]	25	2.76	1.13	27	2.67	1.10	-0.08	[-0.61, 0.46]
Anxiety (20-80)	28	38.21	9.44	30	38.30	11.33	0.01	[-0.50, 0.52]	25	35.24	6.75	27	38.04	12.03	0.28	[-0.26, 0.82]
Openness (1-7)	26	5.50	0.74	30	5.54	0.85	0.05	[-0.47, 0.57]	25	5.64	0.78	27	5.61	0.80	-0.03	[-0.57, 0.51]
Conscientiousness (1-7)	26	5.48	0.98	30	5.47	0.96	-0.01	[-0.53, 0.51]	25	5.63	0.86	27	5.29	1.01	-0.36	[-0.9, 0.18]
Agreeableness (1-7)	26	5.50	0.51	30	5.24	0.91	-0.33	[-0.85, 0.19]	25	5.67	0.63	27	5.39	0.89	-0.35	[-0.89, 0.19]
Extraversion (1-7)	26	4.28	1.01	30	4.50	1.11	0.21	[-0.31, 0.73]	25	4.32	1.04	27	4.59	0.85	0.28	[-0.26, 0.82]
Neuroticism (1-7)	26	3.63	1.17	30	3.38	1.42	-0.18	[-0.70, 0.34]	25	3.54	1.10	27	3.13	1.40	-0.32	[-0.85, 0.22]
Meditation hours	—	—	—	—	—	—	—	—	25	52.95	16.04	27	4.05	2.09	-4.37***	[-5.03, -3.66]
Social Connectedness (1-7)	—	—	—	—	—	—	—	—	25	5.12	1.42	28	5.36	1.52	0.16	[-0.37, 0.69]

The table presents the sample sizes (*N*), means, and standard deviations (*SD*) for each group at each time point along with the effect size (hedge’s g) of between group differences. The dash (—) indicates there were no data collected for these measures at Time 1. g, Hedge’s g; CI, Confidence Interval; Ln, logarithmus naturali or natural log; pg, picograms; ml, milliliter. *p < .05. **p < .01. ***p < .001.

**Table 2:** Within group differences over time.

Variable	Retreat				Control			
	*N*	**Δ**	*g*	*95% CI*	*N*	**Δ**	*g*	*95% CI*
Oxytocin Ln (pg/ml)	24	-0.09	-0.53*	[-0.94, -0.11]	30	0.00	0.01	[-0.34, 0.36]
Vasopressin Ln (pg/ml)	19	-0.47	-0.66	[-1.13, -0.17]	22	-0.17	-0.21	[-0.62, 0.20]
Attachment Anxiety (1-7)	25	-0.22	-0.26	[-0.64, 0.13]	24	-0.07	-0.08	[-0.46, 0.31]
Attachment Avoidance (1-7)	25	0.07	0.08	[-0.30, 0.46]	24	0.02	0.03	[-0.36, 0.41]
Anxiety (20-80)	25	-4.00	-0.46*	[-0.86, -0.06]	26	0.50	0.12	[-0.25, 0.50]
Openness (1-7)	24	0.17	0.47	[0.05, 0.87]	26	0.05	0.11	[-0.26, 0.49]
Conscientiousness (1-7)	24	0.27	0.62*	[0.19, 1.04]	26	-0.11	-0.29	[-0.67, 0.09]
Agreeableness (1-7)	24	0.16	0.37	[-0.04, 0.76]	26	0.10	0.18	[-0.2, 0.56]
Extraversion (1-7)	24	-0.01	-0.01	[-0.40, 0.37]	26	-0.07	-0.15	[-0.52, 0.23]
Neuroticism (1-7)	24	-0.24	-0.35	[-0.74, 0.05]	26	-0.19	-0.37	[-0.76, 0.02]

The table presents the mean change (**Δ**) from Time 1 to Time 2 in each dependent measure for each group. Mean differences are computed using complete cases, so Ns differ from those in **Table 1**. g, Hedge’s g; CI, Confidence Interval; Ln, logarithmus naturali or natural log; pg, picograms; ml, milliliter. *p < .05. **p < .01. ***p < .001.

We also conducted a series of correlation analyses between the hormone and self-report measures. First, we examined associations between oxytocin and vasopressin and the self-report measures of attachment, anxiety, and personality in our full study sample at Time 1. Resultant p-values were corrected for 16 comparisons using a two-stage linear step-up procedure (68). We then used Pearson’s correlations to determine whether the observed changes in oxytocin were predicted by baseline measures of attachment style, anxiety, or personality, or if they were correlated with individual changes in any of these measures over time. We tested these relationships separately for each group and corrected resultant p-values for 32 comparisons using a two-stage linear step-up procedure. Lastly, we assessed the correlations between changes in oxytocin and the amounts of meditation practice undertaken during the study, and between participants’ levels of oxytocin at Time 2 and how personally connected they felt to other meditators in their community.

We did not conduct an a-priori power analysis on oxytocin and vasopressin because our sample size was necessarily constrained by the number of retreat participants who elected to participate in the study and because no prior retreat studies have examined these measures. Instead we conducted a sensitivity power analysis based on the parameters of the collected sample: Given final group sizes of 26 retreatants and 30 controls for oxytocin, we were powered to detect large standardized mean differences (*d* = .74) between groups at Time 2 and medium within-group changes for the retreat (*d* = .57) and control (*d* = .5) groups—assuming two-tailed tests, a significance level of .05, and power of .8. Because the group sizes were smaller for vasopressin (retreat = 20, control = 23), sensitivity analyses indicated we were only powered to detect larger group differences at Time 2 (*d* = .87), and larger within group changes for the retreat (*d* = .66) and control (*d* = .61) groups. Nevertheless, we expected to observe large sized effects given the magnitude and duration of the retreat intervention.

## 3 Results

As reported in Conklin et al. (52), the groups did not significantly differ in age, gender, education, household income, lifetime meditation experience, total number of previous retreats, or longest previously attended retreat (all *p*-values >.05), though retreat participants had attended significantly more days of retreat in the prior year than controls (Control *M* = 10, Retreat *M* = 28, *p* = .016). At Time 1, the groups also did not differ in BMI or measures of health responsibility, physical activity, nutrition, spirituality, or stress management (52). As expected, the retreat group (*n* = 25, *M* = 53 hours, *range* = 14–77 hours) reported practicing significantly more hours of meditation per week than the control group (*n* = 27, *M* = 4.1 hours, *range* = 1–9 hours) during the study.

### 3.1 Oxytocin

**Figure 1:**
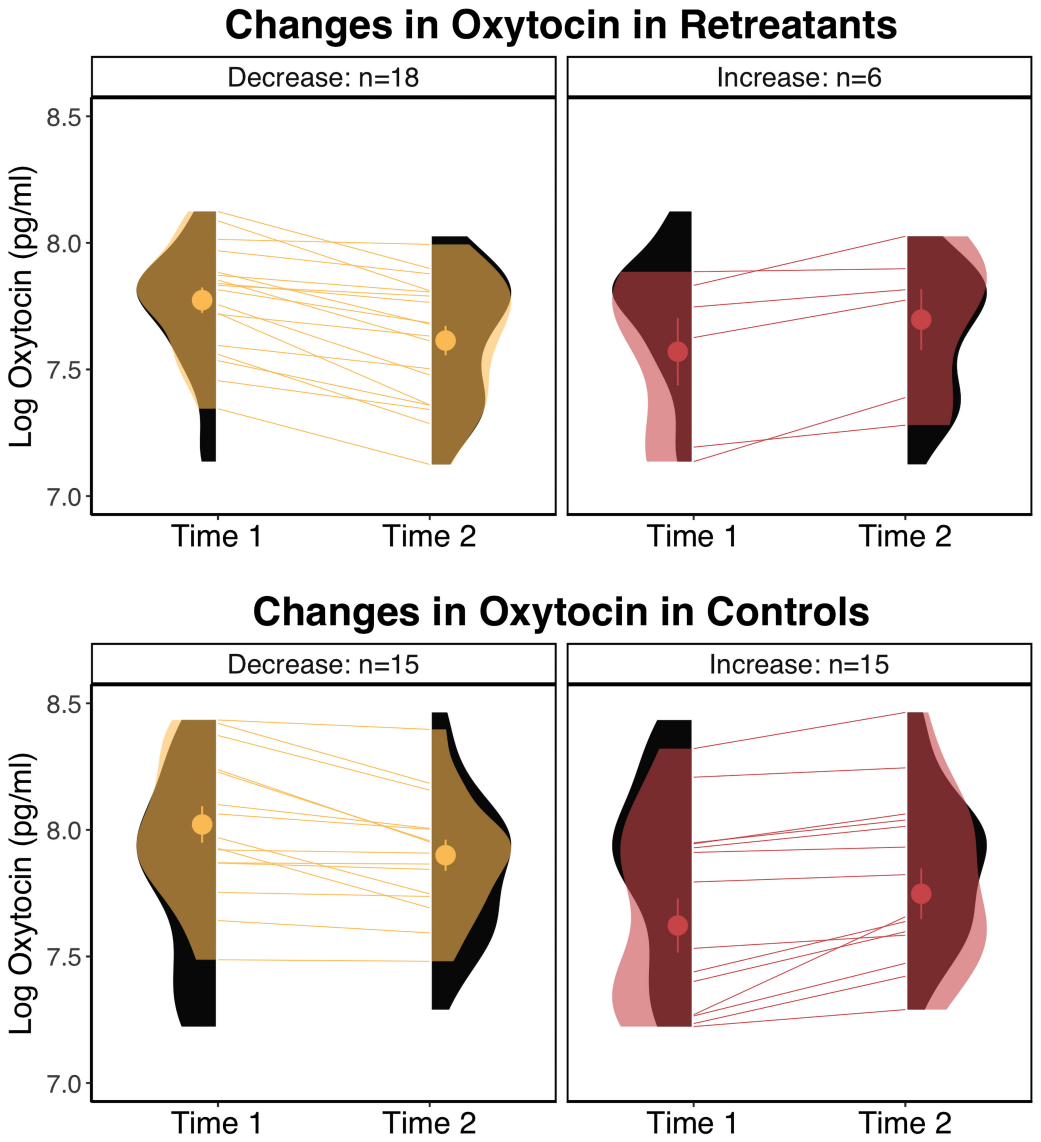
Changes in oxytocin by group. The eyelets represent the mean and standard error of the respective group at the respective time point. The black distributions along each time point depict the overall distribution for the respective group at that time point. The transparent overlays in the panels on the left depict the distributions of participants who decreased within a particular group at a particular time point, and overlays in the panels on the right depict the distributions for participants who increased in oxytocin.  pg/ml, picograms per milliliter.

Summary statistics for all dependent measures are shown in **Table 1** and within group changes are shown in **Table 2**. For oxytocin, there was a significant Group x Time interaction, *F*(1, 52) = 4.61, *p* = .036, but no significant effects of Group, *F*(1, 60) = 2.42, *p* = .125, or Time, *F*(1, 52) = 2.97, *p* = .091. Pairwise comparisons showed a significant decrease in the retreat group from Time 1 to Time 2, *β* = -0.09, *SE* = 0.03, *p* = .042, 95% CI [0, 0.17], but no change in the control group, *β* = 0.01, *SE* = 0.03, *p* = .997, 95% CI [-0.09, 0.07]. There were no significant differences between the retreat and control group at Time 1, *β* = -0.08, *SE* = 0.08, *p* = .777, 95% CI [-0.14, 0.29], or Time 2, *β* = -0.17, *SE* = 0.08, *p* = .167, 95% CI [-0.05, 0.39]. Changes in oxytocin by group are presented in **Figure 1**.

**Figure 2:**
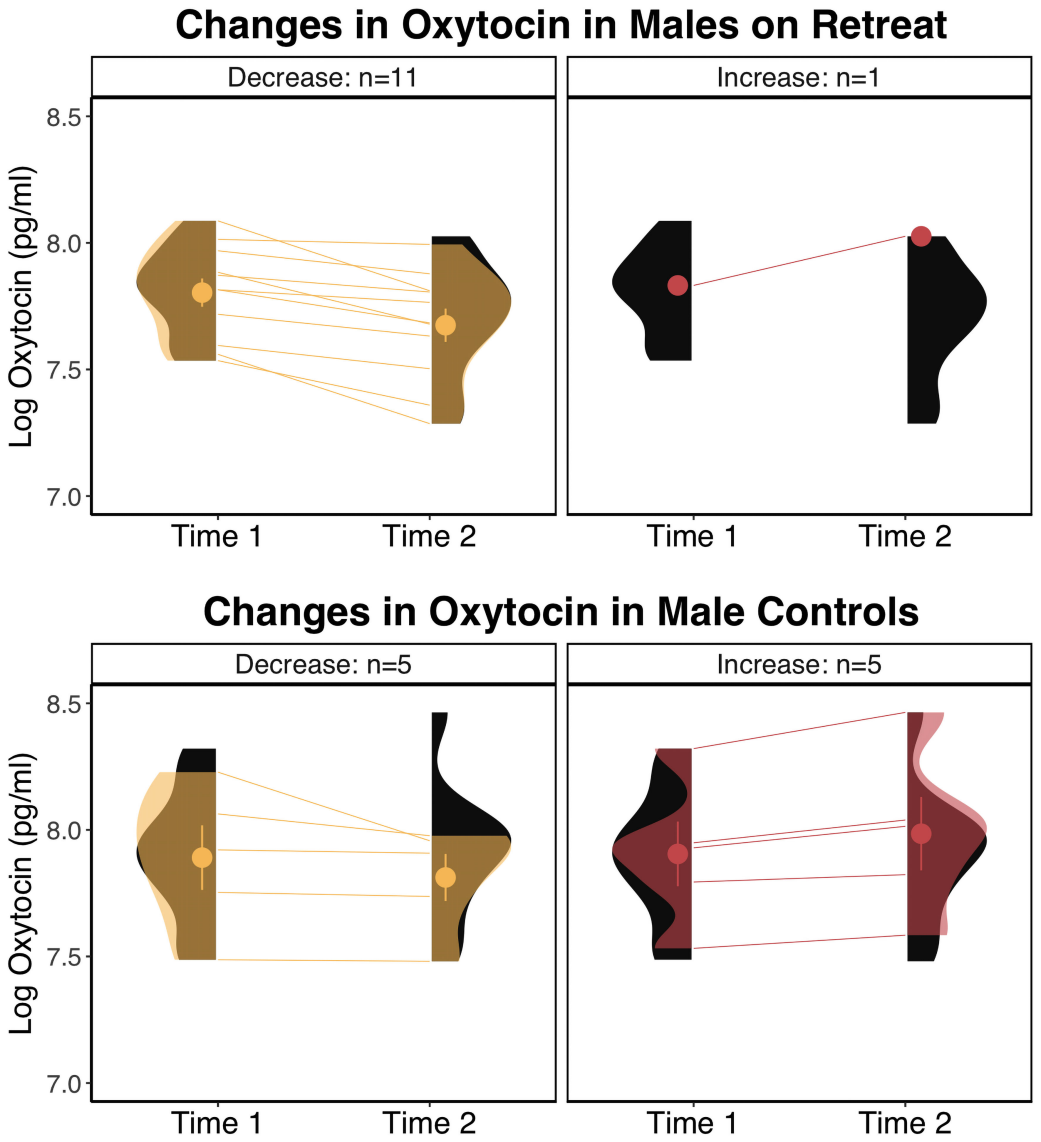
Changes in log transformed oxytocin levels in males by group. The eyelets represent the mean and standard error of the respective group at the respective time point. The black distributions along each time point depict the overall distribution for the respective group at that time point. The transparent overlays in the panels on the left depict the distributions of participants who decreased within a particular group at a particular time point, and overlays in the panels on the right depict the distributions for participants who increased in oxytocin. pg/ml, picograms per milliliter.

When we tested the effects of retreat separately for males and females, the model for females showed no significant Group x Time interaction, *F*(1, 29) = .79, *p* = .383, and no significant effects of Group, *F*(1, 35) = 1.78, *p* = .191, or Time, *F*(1, 29) = .23, *p* = .634. For males, the Group x Time interaction was on the cusp of significance, *F*(1, 20) = 4.11, *p* = .056, and there were no significant effects of Group, *F*(1, 22) = 1.66, *p* = .211, or Time, *F*(1, 20) = 3.71, *p* = .069. For males, pairwise comparisons revealed a pattern similar to the overall analyses, with retreat participants showing a nearly significant decrease in oxytocin, *β* = -0.1, *SE* = 0.04, *p* = .051, 95% *CI* [-0.2, 0], compared to controls who showed no change, *β* = 0, *SE* = 0.04, *p* = .999, 95% CI [-0.1, 0.11]. There were no differences between males in the retreat and control groups at Time 1, *β* = -0.07, *SE* = 0.1, *p* = .876, 95% CI [-0.34, 0.2], or Time 2, *β* = -0.18, *SE* = 0.1, *p* = .295, 95% CI [-0.45, 0.1]. In **Figure 2**, we compare changes in oxytocin in males by group, noting that the proportion of male retreat participants who decreased (11 out of 12) was much higher than control males (5 out of 10) or females on retreat (6 out of 11; not shown).

### 3.2 Vasopressin

For vasopressin, there was no significant Group x Time interaction, *F*(1, 39) = 1.04, *p* = .314, and no significant effect of Group, *F*(1, 50) = 0.35, *p* = .558. There was a significant effect of Time, *F*(1, 39) = 6.73, *p* = .013. Pairwise comparisons indicated that both groups decreased, but that this change was not significant in either group separately, and that the groups did not differ at Time 1 or Time 2 (all *p*-values >.05). Given the lack of significant retreat effects and the number of samples that were undetectable, we did not test further associations between vasopressin and self-report measures.

### 3.3 Self-report measures

As reported in Conklin et al. (52), we observed significant decreases in anxiety and increases in conscientiousness in the retreat group over time, but there were no group differences or retreat-related changes in extroversion, agreeableness, neuroticism, or openness to experience (effect sizes shown in **Tables 1**, **2**). We also found no significant effects of Group, Time, or Group x Time interactions for attachment anxiety and avoidance (all Group x Time interaction *p*-values >.05).

### 3.4 Associations between hormone and self-report measures

When we examined correlations between hormone and self-report measures across groups at Time 1, we found a significant positive correlation between oxytocin and attachment anxiety, *r*(53) = 0.27, p = .042, *p_adj_* = .405—though this association did not survive correction for multiple comparisons. We found no other correlations between oxytocin or vasopressin and attachment avoidance, anxiety, agreeableness, conscientiousness, extraversion, neuroticism, or openess at Time 1 (all *p*-values >. 05).

**Figure 3:**
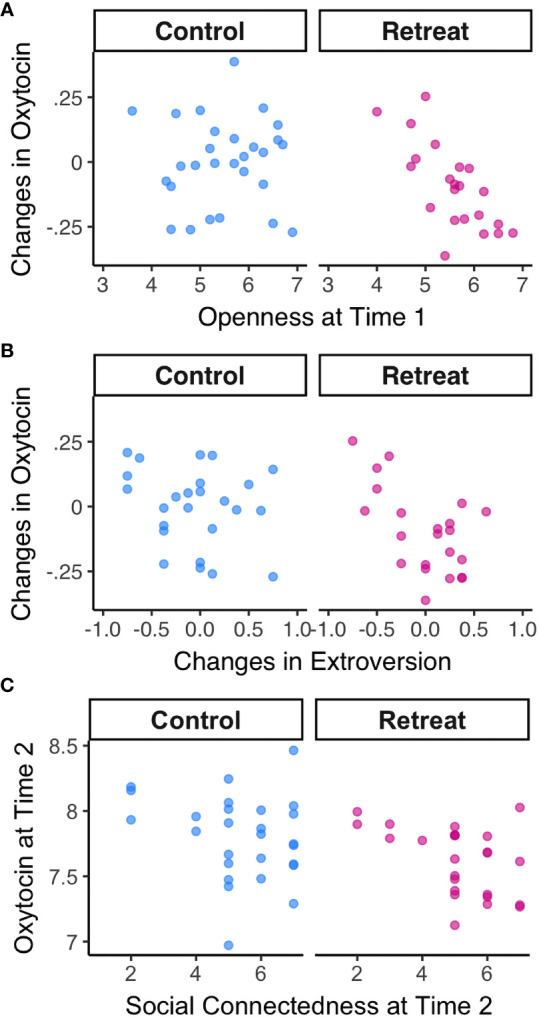
Correlations between oxytocin and psychosocial metrics by Group. **(A)** Correlations between openness to experience at Time 1 and changes in oxytocin; **(B)** Correlations between changes in oxytocin and changes in extroversion; **(C)** Correlations between oxytocin and feelings of personal connection with fellow meditators at Time 2.

Next, we examined relationships between baseline self-report variables and changes in oxytocin over time. We found a strong negative correlation between openness to experience at Time 1 and changes in oxytocin in the retreat group, *r*(20) = -0.72*, p* <.000, *p_adj_* = .004, but not controls, *r*(26) = 0.01*, p* = .962, *p_adj_* = .991 (depicted in **Figure 3A**). There were no significant correlations between changes in oxytocin and baseline attachment avoidance, attachment anxiety, trait anxiety, extroversion, neuroticism, agreeableness, or conscientiousness (all *p*-values >. 05).

When we examined changes in each self-report predictor in relation to changes in oxytocin, we found a significant negative correlation between changes in extroversion and changes in oxytocin for retreatants, *r*(20) = -0.58*, p* = .005, *p_adj_* = .079, such that decreases in oxytocin were associated with increases in extroversion—though this association did not survive correction for multiple comparisons (depicted in **Figure 3B**). There was no such correlation in controls, *r*(23) = -0.23*, p* = .271, *p_adj_* = .830. There were no significant associations between changes in oxytocin and changes in any other self-report measures (all *p*-values >.05).

Finally, we found no correlation between hours of meditation practiced during the study and changes in oxytocin in the retreat group, *r*(21) = .11, *p* = .634, or controls, *r*(24) = -.04, *p* = .864. We did, however, find a significant correlation between oxytocin levels at Time 2 and how personally connected retreat participants felt to their meditation community, *r*(23) = -0.47, *p* = .019 (depicted in **Figure 3C**). The association between oxytocin and feelings of connection at Time 2 was not significant for controls, *r*(25) = -0.23, *p* = .25.

## 4 Discussion

We investigated changes in oxytocin and vasopressin during a month-long Insight meditation retreat, as well as their relation to measures of attachment style, anxiety, personality, and social connectedness. We found a small but significant decrease in oxytocin in the meditation retreat group, with no significant change in controls, suggesting that silent retreat practice may elicit a reduction in oxytocin production. We also found a significant decrease in vasopressin across both groups, suggesting no specific effect of retreat. However, the vasopressin concentration in many of our samples fell below the level of detection, which limited our available sample and ability to interpret these results. In the retreat group, higher openness to experience at Time 1 predicted greater reductions in oxytocin during the retreat, and lower oxytocin at Time 2 was related to stronger feelings of personal connection with fellow meditators in the retreat group. The changes in oxytocin were not related to attachment style or trait anxiety.

At first glance, the observed decrease in oxytocin during retreat may seem at odds with established accounts of oxytocin’s involvement in prosocial and affiliative processes (69). However, there is a growing consensus that many of the findings regarding oxytocin and social behavior are person- and context-dependent (70). Several additional theories have been proposed to synthesize this extensive but often disparate literature, including anxiolytic and stress-related hypotheses (30, 71–73); the in-/out-group hypothesis (74, 75); the social salience hypothesis (76); the general approach−avoidance hypothesis (77, 78); and the allostatic theory of oxytocin (79). In the following sections, we interpret our findings from each of these perspectives—highlighting aspects of the meditation training and retreat intervention throughout.

### 4.1 Prosocial/affiliative hypothesis

The primary focus of the retreat intervention was mindfulness meditation, which included practice instructions to support awareness of one’s present moment experience and to relate to that experience without grasping, aversion, or delusion (56, 80). By developing these capacities, such training is expected to promote loving-kindness, which is conceptualized as a form of inclusive caring that recognizes the interconnectedness of all living beings (80). The retreat also included instruction in loving-kindness, compassion, empathetic joy, and equanimity. These meditation practices often involve guided imagery or the repetition of phrases meant to evoke feelings of care toward oneself and others. Such practices are intended to develop a prosocial orientation toward others and may function as an internal simulation or manifestation of social support (81–83).

In line with the aims of this training, we expected that oxytocin levels would increase in the retreat group and would be associated with improvements in attachment security and feelings of social connectedness. Although we did not find general support for these hypotheses, we did observe a relationship between oxytocin and social connectedness following retreat training—though the direction of this relationship suggests that lower oxytocin was related to greater feelings of connection. Additionally, the observed relationship between changes in oxytocin and changes in extroversion might suggest that individuals who demonstrated oxytocin reductions may have also experienced an increased desire for social engagement after weeks of silent retreat. However, this relationship did not survive correction for multiple comparisons. Moreover, contrary to our expectations, we observed no relationships between oxytocin and measures of attachment style, and no increases in secure attachment during retreat, suggesting that our findings may not necessarily represent changes in underlying patterns of attachment. This interpretation is consistent with a recent meta-analysis that found no relationship between endogenous oxytocin and attachment security (84). It is unclear, however, whether a different pattern of results would have emerged if the retreat was focused exclusively on relational practices including loving-kindness and compassion meditation. Research is needed that directly compares oxytocin outcomes from retreat interventions with different training emphases.

### 4.2 Anxiolytic/stress hypotheses

The prediction that oxytocin may be involved in meditation induced relaxation responses and prosocial behavior (1, 2) is, in part, predicated on the assumption that elevated oxytocin reduces stress and anxiety, which in turn may promote more prosocial engagement (76). Based on this hypothesis, we expected to see increases in oxytocin across the course of retreat and thought that these increases may be associated with reductions in anxiety. Instead we observed decreases in oxytocin that were unrelated to the decreases in anxiety observed. 

A closer review of the research on oxytocin, stress responding, and anxiety suggests that there are important nuances in this literature that may better situate our findings. For example, the ‘tend-and-befriend’ theory posits that under threat, elevated oxytocin levels may motivate women to seek out social support and may, therefore, be interpreted as a marker of distress (66, 68). In support of this idea, elevated peripheral oxytocin levels have been observed across instances of relational distress, anxiety, and depression (although the opposite has also been found—reviewed in 7). Thus, the reductions in oxytocin observed in our study may represent reductions in social distress while on retreat. This interpretation is consistent with our finding that lower levels of oxytocin were related to greater feelings of social connection at the end of retreat, and the reductions in anxiety that we observed in the retreat group. It is interesting to note, however, that the relationships between elevated oxytocin and distress have predominately been found in women, whereas the reductions in oxytocin observed in this study primarily occurred in the male retreat participants.

Given the short half-life of oxytocin in blood (2–8 minutes) (59, 79, 85, 86), another possibility is that the lower levels of oxytocin at Time 2 represent a decrease in stress reactivity to the blood draw following weeks of meditation training. While we cannot directly assess this assumption in our retreat group, data from our control group suggests that subjective experiences of stress related to the blood draw and leading up to the blood draw were not associated with oxytocin levels at either time point ^^5^^.

### 4.3 In-/out-group hypothesis

As evidence emerged that oxytocin’s effects on social behavior and cooperation were not uniformly positive, De Dreu and colleagues formulated the in-/out-group hypothesis to better account for these inconsistencies (74, 75). This theory suggests that oxytocin regulates cooperation and conflict in social species in the context of intergroup relations—increasing positive concern for familiar individuals over the interests of genetically or culturally unfamiliar out-group members (87). In support of this theory, they have demonstrated that oxytocin administration can promote ethnocentrism (88), parochial altruism (89), and within-group coordination of out-group attacks (90).

In the context of meditation training, the oxytocin system is an interesting biological target with respect to this in-/out-group framing, as many forms of meditation are intended to promote compassion and prosocial attitudes toward an increasingly broad and more inclusive range of targets, including friends, acquaintances, strangers, difficult others, and eventually all sentient beings (82). Given its involvement in prosocial processes like empathy and trust, elevated oxytocin has been expected to promote or facilitate compassion and loving-kindness. However, it may be that elevated oxytocin presents a barrier to compassion by making the distinction between in-group and out-group identity more salient. If this is the case, retreat-related reductions in oxytocin may facilitate the development of prosocial orientations toward those we view as more different from ourselves. Research examining the effects of intranasal oxytocin administration on compassionate responses to the various relational targets outlined in traditional meditation instructions could inform this point.

### 4.4 Social salience hypothesis

In an effort to reconcile the prosocial, anxiolytic/stress, and in-/out-group hypotheses, other investigators proposed the social salience hypothesis (70, 76). This theory posits that oxytocin modulates the perceptual salience and processing of social cues by regulating dopamine’s salience coding and attention reorienting effects (76). In an extension of this theory, Steinman, Duque-Wilckens and Trainor (91) review literature suggesting that there are overlapping but distinct neural circuits that mediate the effects of oxytocin in appetitive and aversive social contexts—with the nucleus accumbens and ventral tegmental area facilitating social reward and approach behavior, and the bed nucleus of the stria terminalis mediating avoidance in socially aversive contexts (91).

In the retreats studied here, we may expect increased orientation toward social cues given that participants lived onsite with teachers and 80 to 100 fellow practitioners, most of whom are strangers. However, the retreats also involved a set of behavioral guidelines that may have reduced practitioners’ need to attend to typical social cues. For example, the retreats began with a ritual on the five precepts, in which participants agree to refrain from 1) taking life (e.g., killing), 2) taking what is not freely given (e.g., stealing), 3) misuse of the senses (often related to sexual behavior), 4) wrong speech (e.g., lying), and 5) intoxicants for the course of the retreat. These precepts are intended to facilitate a physically, socially, and psychologically safe environment to support sustained focus on the practice. Retreat participants also practiced noble silence, during which they avoided deliberately engaging with or conversing with others, despite spending time in close physical proximity (92). This unique social context may reduce social demands while preserving some advantages of social proximity—including a state of calm or equanimity, supported by bodily synchronization and emotional attunement (92). Additionally, oxytocin is released in response to many social behaviors that are intentionally renounced during retreat—including affiliative vocalizations, eye contact, social touch, and sexual behavior (93–95). As such, the withdrawal from these normative forms of social interaction may have contributed to the observed decreases in oxytocin while on retreat.

### 4.5 General approach−avoidance hypothesis

The general approach−avoidance hypothesis challenges the notion that the oxytocin system is socially specific—arguing instead that oxytocin may regulate approach and avoidance behavior in a generalized way (77, 78, 96). Harari-Dahan and Bernstein (77) note that the neural substrates for social and non-social approach and avoidance are the same and argue that oxytocin enhances the salience of personally meaningful and emotionally evocative stimuli, regardless of whether they are social or non-social (77).

Considered through this lens, the retreat-related reductions in oxytocin may indicate a shift in appetitive and avoidant motivations as a function of meditation practice and the retreat context. Insight meditation practice involves closely observing, and actively inhibiting, impulses to approach or avoid internal and external stimuli (e.g., engaging in a ruminative pattern of thought or greeting people as you enter a room). Moreover, the retreat environment is designed to limit and adjust the kinds of external stimuli practitioners engage with in order to promote attention to internal experiences, which is believed to facilitate insight and shifts in one’s relationship to experience. For example, at mealtime practitioners might be instructed to notice sensations related to hunger, and to observe how those sensations change as they walk to the dining hall, when they take their first bite, and as they reach or pass satiation. Through such observation, practitioners may begin to better understand, and potentially modulate, their conditioned responses to food cues. Consequently, the observed reductions in oxytocin may reflect shifts in the relative salience and prioritization of both social and non-social stimuli in the environment. This interpretation is consistent with evidence that mindfulness-based interventions can help to reduce habitual reward responses to drug-related cues while enhancing responses to healthy rewards in those experiencing opioid addiction (97, 98). Nevertheless, our findings seem to contradict research suggesting that greater plasma oxytocin is related to less craving in former heroine users (99).

### 4.6 The allostatic theory of oxytocin

Finally, the allostatic theory of oxytocin situates the social functions of oxytocin within a broader framework of anticipatory and survival related functions (79). According to this theory, oxytocin mediates the sensing, learning, predicting, and responding necessary for allostatic regulation, including shifting physiological set points in response to temporal and local conditions. In support of this model, when Quintana et al. (100) compared the distribution of oxytocin receptor expression in the brain to cognitive state maps generated from over 14,000 fMRI studies, they found that the oxytocin receptor gene was among the top .05% of genes most strongly associated with anticipatory, appetitive, and aversive states out of over 20,000 protein-coding genes (100). Rather than diminish the social functions of oxytocin, this perspective situates social behavior as a critical mechanism of adaptation for social species that is integrated with many physiological systems throughout the body. This view helps to explain oxytocin’s involvement in processes like digestion (101), metabolism (102), the immune system (103), and cardiovascular function (104).

If considered from this lens, the decreases in oxytocin might reflect an adaptation to the relative stability of the retreat environment and routine. In this context, the association we observed between openness to experience and oxytocin reductions may represent a form of malleability that promotes adaptation to the retreat environment. In a residential retreat, lodging and regular meals are provided, and most hours of the day are scheduled. This structure is designed to support mental stability and calm, allowing access to increasingly subtler sensations and experiential states. Thus, retreats may represent an important opportunity for the brain and body to update certain predictive models, with the intention of recognizing and potentially adjusting maladaptive patterns. Importantly, the allostatic theory of oxytocin also provides a framework for understanding how meditation or mindfulness-related modulation of the oxytocin system might contribute to broader scale health improvements.

### 4.7 Strengths and limitations

To our knowledge, this is the first longitudinal study to measure endogenous oxytocin in the context of a residential meditation retreat. The advantages of this design include the high dose of meditation practice and the ecological validity of the retreat, which has been offered annually at Spirit Rock for decades. Moreover, our control group was composed of similarly experienced meditators who had themselves attended intensive retreats, which helps to minimize the chance that the differences we observed were principally the result of lifestyle differences between regular meditation practitioners and non-meditators.

Despite these strengths, our design prevents us from distinguishing the effects of meditation practice from the broader social, behavioral, and situational changes inherent to retreats. However, all of these components are likely synergistic aspects of the intervention, and our goal was to assess the retreat intervention as a whole. Additionally, coordinating with the retreat center to maintain the integrity of the retreat imposed logistical constraints that limited our available sample size and precluded a true pre-retreat assessment and randomization to groups. The generalizability of our findings is also limited by participants’ self-selection into the retreat and study, as residential retreats are often only accessible to people with the means and flexibility to leave their home and jobs for extended periods of time.

Another limitation of our design was the asynchronous assessment of retreat participants and controls. Retreat participants were assessed over a 2-month period, whereas controls were assessed across an entire year. Animal studies indicate there is seasonal variation in oxytocin and oxytocin receptor expression in species that demonstrate seasonally specific mating and group behavior (105). In humans, we expect these patterns to be more complex given the range of environments and behavioral patterns we have adapted to, though there is little empirical research on this topic. Preliminary evidence does, however, suggest that there may be seasonal variations (106) that could have contributed more variability in our control group. It might also be that the retreat effects in women were obscured by fluctuations in oxytocin related to their menstrual cycles. Oxytocin levels have been shown to fluctuate with estrogen levels (107, 108), so menstrual cycle stage and estrogen levels will be important covariates to include in future studies.

Another important limitation of this study is the measurement and interpretation of plasma oxytocin and vasopressin. We measured the concentration of these hormones in raw plasma using ELISA assays without the extraction step. There are several compelling arguments against the assessment of oxytocin in unextracted samples, which suggest that resultant oxytocin values may be grossly overestimated, contaminated by other molecular species, or even completely arbitrary (59, 60, 109). On the other hand, mass-spectrometry results suggest that plasma oxytocin levels may be higher than previously understood, and that the discrepancies between raw and extracted samples may represent the distinction between free and total oxytocin (61). Brandtzaeg and colleagues (61) proposed that the majority of endogenous plasma oxytocin is bound to other proteins, which are eliminated during the extraction process. If this is the case, then free and bound fractions of oxytocin may have different implications for behavior; however, the biological activity of bound oxytocin remains unclear. Interestingly, advocates of extraction also note that both extracted and non-extracted samples seem to contain immunoreactive molecules other than oxytocin (59, 60, 110). It is hypothesized that some of these molecular species may be oxytocin degradation products, and therefore still reflective of the oxytocin system (60, 109). Unfortunately, comparative analyses suggest that ELISA kits from Enzo Life Sciences may be particularly affected by immunoassay interference (62).

It is also worth noting the range of peptide values observed in this study. Many of our vasopressin samples fell below the lower limit of detection for this assay, suggesting that plasma vasopressin may not be a viable measure in healthy humans. Meanwhile, the oxytocin values observed were particularly high, with many of our samples exceeding the upper limit of the assay curve. Although unextracted samples are expected to have higher concentrations than extracted samples samples, the range of raw oxytocin values observed in this study (933–4623 pg/ml) was considerably higher than other studies reporting mean values in the lower hundreds—though some studies with unextracted human plasma samples have reported similar values (e.g., 111). Moreover, we know from communications with Enzo that the company had recently transitioned to a new oxytocin assay and antibody, meaning that these values may not be directly comparable to those generated from previous versions of the assay kit. Given these limitations, many of which are discussed in greater detail by Tabak and colleagues (34), it is critical that our findings be replicated with more reliable measurement methods.

### 4.8 Future directions

Our findings raise the possibility that oxytocin production may decline as the result of intensive meditation practice as well as the reduced social interaction characteristic of silent meditation retreats. This raises interesting implications to be tested in future studies. For example, reductions in oxytocin during a silent retreat may signal a sensitivity or openness to subsequent social interactions following social withdrawal. That is, the prolonged period of reduced social interaction may serve to re-sensitize an individual to social exchanges, such that they may be more attuned to social cues immediately following retreat. This hypothesis is in line with theory and data suggesting that meditation training may function to enhance the motivational salience of interpersonal cues, particularly those relevant to processes of empathy and compassion (50). In addition to measuring hormone levels at multiple time points throughout retreat, future studies would benefit from including follow-up assessments to investigate oxytocin fluctuations and social engagement after retreat. It will also be useful to compare different styles of meditation interventions. For example, comparing a silent meditation retreat to a relational retreat, which includes periods of guided social interaction in addition to silent meditation practice, would help to determine whether the reductions in oxytocin are driven by the reductions in social interaction or other aspects of the retreat intervention.

Additionally, considering our results alongside the two studies that found increased oxytocin during acute meditation sessions (i.e., 40–42) suggests that short-term practice and more intensive training may carry different biological signatures or trajectories, and that the practice context may be particularly influential. Future studies will be needed to understand how patterns shift as practitioners develop meditative experience and practice across different contexts.

In addition to circulating oxytocin levels, it will also be important for future studies to consider oxytocin genotypes and receptor expression. For example, in the first study to link meditation to the oxytocin system, Isgett et al. (112) found that a polymorphism of the oxytocin receptor gene rs1042778 moderated the effects of a 6-week loving-kindness program versus a mindfulness meditation program on participants’ positive emotions. Participants in the loving-kindness condition with the GG allele—which has been linked to lower negative affectivity and less inhibited sociality—demonstrated greater increases in positive emotions than those without the GG allele or those who participated in the mindfulness meditation condition (112). As such, it will likely be necessary to track more facets of the oxytocin system to fully understand the effects of meditation on oxytocin signaling.

Finally, although there were no statistically significant differences between genders in our study, it is worth noting that the pattern of oxytocin reduction was more consistently observed in males (11 out of 12 participants) than in females (6 out of 12) on retreat. This finding warrants replication in a larger sample to determine if there are sex differences in the physiological benefits of practicing in an intensive retreat context. To date, there is limited evidence suggesting that there are sex differences in the effects of intranasal oxytocin on empathy. A recent meta-analysis found that there was no statistically significant difference between sexes; however, the authors noted that in studies with males only, the effect of intranasal oxytocin was consistently positive, whereas the effects in studies that included females were close to zero (26). They hypothesize that females may have higher baseline empathic abilities due to differences in endogenous oxytocin levels and the degree to which women are socialized to express empathy, which is consistent with the finding that intranasal oxytocin increased the empathic responses in males to a level similar to that of untreated females (113). Future studies will be needed to assess these potential differences in relation to retreat practice.

## 5 Conclusions

Our data provide compelling alternatives to the prosocial and attachment-mediated hypotheses regarding oxytocin and meditation, suggesting that oxytocin may decrease during intensive meditation retreats. Given the comprehensive nature of the retreat intervention and the pleiotropic functions of oxytocin, it is plausible that this finding reflects broad-scale changes in approach/avoidance behavior, reward processing, and allostatic regulatory functions, which may ultimately contribute to changes in social behavior and overall well-being. To extend this work, we advocate for more nuanced study designs that assess multiple aspects of the oxytocin system while also differentiating between the potential effects of different practice types and contexts.

## Data availability statement

The raw data supporting the conclusions of this article will be made available by the authors, without undue reservation.

## Ethics statement

The studies involving humans were approved by University of California, Davis Institutional Review Board. The studies were conducted in accordance with the local legislation and institutional requirements. The participants provided their written informed consent to participate in this study.

## Author contributions

QC: Data curation, Formal analysis, Investigation, Project administration, Visualization, Writing – original draft, Writing – review & editing. BK: Conceptualization, Investigation, Writing – review & editing, Data curation. AZ: Conceptualization, Investigation, Writing – review & editing. EE: Resources, Supervision, Writing – review & editing. CS: Conceptualization, Funding acquisition, Investigation, Methodology, Project administration, Resources, Supervision, Writing – review & editing.

## Footnotes

1 For some participants, domestic or international travel was required to attend the retreat, which made blood collection prior to the onset of the retreat infeasible. Additionally, given the potential stress of travel, retreatants were given a full day to acclimate to the retreat environment before the first assessment. Therefore, the Time 1 measures were not strictly a pre-intervention baseline.

2 This meditation session was included to allow control participants to acclimate to the retreat center after driving from their homes, given that retreat participants were not required to travel.

3 For arguments in favor of extraction see Leng and Sabatier (59) and Szeto et al. (60); for arguments against extraction, see Brandtzaeg et al. (61) and Gnanadesikan et al. (62).

4 The intra-class correlation coefficients were calculated using the ‘rpt’ function from the *rptR* package. Models were fitted on the log-transformed hormone data, as described in the Data Processing and Statistical Analyses section using the *lme4* package. To account for the study design, the models included a random intercept for participant and fixed effects for Group, Time, and the Group x Time interaction, as recommended by the telomere research network (https://trn.tulane.edu/wp-content/uploads/sites/445/2020/10/How-to-calculate-repeatability.pdf).

5 At each assessment, the control group filled out a brief stress questionnaire (7 items) regarding their subjective experience of the blood draw as well as their perceived stress in the days, weeks, and months leading up to the blood draw. We tested the correlation between log transformed oxytocin and each of these questions, as well as the average of these questions, and FDR corrected the resultant *p*-values. There were no significant correlations (-0.39 < *rs* < 0.23, adjusted *p*-values > .05).

## References

[1] Mascaro JS Darcher A Negi LT Raison CL. The neural mediators of kindness-based meditation: A theoretical model. Front Psychol. (2015) 6:109. doi: 10.3389/fpsyg.2015.00109 25729374 PMC4325657

[2] Luberto CM Shinday N Song R Philpotts LL Park ER Fricchione GL. A systematic review and meta-analysis of the effects of meditation on empathy, compassion, and prosocial behaviors. Mindfulness. (2018) 9:708–24. doi: 10.1007/s12671-017-0841-8 PMC6081743 30100929

[3] McCook O Denoix N Merz T. The Gasotransmitter Hydrogen Sulfide and the Neuropeptide Oxytocin as Potential Mediators of Beneficial Cardiovascular Effects through Meditation after Traumatic Events. Trauma Care. (2021) 1(3):183–94. doi: 10.3390/traumacare1030016

[4] Rigney N de Vries GJ Petrulis A Young LJ. Oxytocin, vasopressin, and social behavior: from neural circuits to clinical opportunities. Endocrinology. (2022) 163:bqac111. doi: 10.1210/endocr/bqac111 35863332 PMC9337272

[5] Gimpl G Fahrenholz F. The oxytocin receptor system: structure, function, and regulation. Physiol Rev. (2001) 81:629–83. doi: 10.1152/physrev.2001.81.2.629 11274341

[6] Grinevich V Neumann ID. Brain oxytocin: How puzzle stones from animal studies translate into psychiatry. Mol Psychiatry. (2021) 26:265–79. doi: 10.1038/s41380-020-0802-9 PMC7278240 32514104

[7] Takayanagi Y Onaka T. Roles of oxytocin in stress responses, allostasis and resilience. Int J Mol Sci. (2022) 23(1). doi: 10.3390/ijms23010150 PMC8745417 35008574

[8] Manning M Stoev S Chini B Durroux T Mouillac B Guillon G. Peptide and non-peptide agonists and antagonists for the vasopressin and oxytocin V1a, V1b, V2 and OT receptors: Research tools and potential therapeutic agents. Progress in Brain Research. Elsevier (2008). p. 473–512. doi: 10.1016/S0079-6123(08)00437-8 18655903

[9] Carson DS Guastella AJ Taylor ER McGregor IS. A brief history of oxytocin and its role in modulating psychostimulant effects. J Psychopharmacol. (2013) 27:231–47. doi: 10.1177/0269881112473788 23348754

[10] Bridges RS. The behavioral neuroendocrinology of maternal behavior: Past accomplishments and future directions. Hormones Behav. (2020) 120:104662. doi: 10.1016/j.yhbeh.2019.104662 PMC7117973 31927023

[11] Scatliffe N Casavant S Vittner D Cong X. Oxytocin and early parent-infant interactions: A systematic review. Int J Nurs Sci. (2019) 6:445–53. doi: 10.1016/j.ijnss.2019.09.009 PMC6838998 31728399

[12] Numan M Young LJ. Neural mechanisms of mother-infant bonding and pair bonding: Similarities, differences, and broader implications. Hormones Behav. (2016) 77:98–112. doi: 10.1016/j.yhbeh.2015.05.015 PMC4671834 26062432

[13] Bales KL del Razo RA Conklin QA Hartman S Mayer HS Rogers FD. Titi monkeys as a novel non-human primate model for the neurobiology of pair bonding. Yale J Biol Med. (2017) 90(3):373–87. PMC5612182 28955178

[14] Rigney N de Vries GJ Petrulis A. Modulation of social behavior by distinct vasopressin sources. Front Endocrinol. (2023) 14:1127792. doi: 10.3389/fendo.2023.1127792 PMC9968743 36860367

[15] Cuzzo B Padala SA Lappin SL. Physiology, vasopressin. In: StatPearls. Treasure Island (FL): StatPearls Publishing (2023). Available at: http://www.ncbi.nlm.nih.gov/books/NBK526069/. 30252325

[16] Piper WT Saturn SR. Social neuropeptides and behavior. In: Encyclopedia of Mental Health. Elsevier, Cambridge, Massachusetts (2016). p. 184–8. doi: 10.1016/B978-0-12-397045-9.00046-X

[17] Kelly AM Goodson JL. Functional significance of a phylogenetically widespread sexual dimorphism in vasotocin/vasopressin production. Hormones Behav. (2013) 64:840–6. doi: 10.1016/j.yhbeh.2013.09.006 24100197

[18] Caldwell HK. Oxytocin and sex differences in behavior. Curr Opin Behav Sci. (2018) 23:13–20. doi: 10.1016/j.cobeha.2018.02.002

[19] Lieberz J Scheele D Spengler FB Matheisen T Schneider L Stoffel-Wagner B. Kinetics of oxytocin effects on amygdala and striatal reactivity vary between women and men. Neuropsychopharmacology. (2020) 45:1134–40. doi: 10.1038/s41386-019-0582-6 PMC7235226 31785587

[20] Macdonald K Macdonald TM. The peptide that binds: A systematic review of oxytocin and its prosocial effects in humans. Harvard Rev Psychiatry. (2010) 18:1–21. doi: 10.3109/10673220903523615 20047458

[21] Kosfeld M Heinrichs M Zak PJ Fischbacher U Fehr E. Oxytocin increases trust in humans. Nature. (2005) 435:673–676). doi: 10.1038/nature03701 15931222

[22] Zak PJ Kurzban R Matzner WT. Oxytocin is associated with human trustworthiness. Hormones Behav. (2005) 48:522–7. doi: 10.1016/j.yhbeh.2005.07.009 16109416

[23] Zak PJ Stanton A Ahmadi S. Oxytocin increases generosity in humans. PloS One. (2007) 2:1–5. doi: 10.1371/journal.pone.0001128 PMC2040517 17987115

[24] Barraza JA Zak PJ. Empathy toward strangers triggers oxytocin release and subsequent generosity. Ann New York Acad Sci. (2009) 1167:182–9. doi: 10.1111/j.1749-6632.2009.04504.x 19580564

[25] Bartz JA Zaki J Bolger N Hollander E Ludwig NN Kolevzon A. Oxytocin selectively improves empathic accuracy. psychol Sci. (2010) 21:1426–8. doi: 10.1177/0956797610383439 PMC6634294 20855907

[26] Stark N Bobadilla L Michael P Saturn S Portner M. A meta-analytic review of the relationship between empathy and oxytocin: Implications for application in psychopathy research. Aggression Violent Behav. (2023) 70:101828. doi: 10.1016/j.avb.2023.101828

[27] Akinrinade I Kareklas K Teles MC Reis TK Gliksberg M Petri G. Evolutionarily conserved role of oxytocin in social fear contagion in zebrafish. Science. (2023) 379:1232–7. doi: 10.1126/science.abq5158 36952426

[28] Tops M van Peer JM Korf J Wijers A Tucker DM. Anxiety, cortisol, and attachment predict plasma oxytocin. Psychophysiology. (2007) 44:444–9. doi: 10.1111/j.1469-8986.2007.00510.x 17371496

[29] Buchheim A Heinrichs M George C Pokorny D Koops E Henningsen P. Oxytocin enhances the experience of attachment security. Psychoneuroendocrinology. (2009) 34:1417–22. doi: 10.1016/j.psyneuen.2009.04.002 PMC3138620 19457618

[30] Hostinar CE Sullivan RM Gunnar MR. Psychobiological mechanisms underlying the social buffering of the hypothalamic-pituitary-adrenocortical axis: A review of animal models and human studies across development. psychol Bull. (2014) 140:256–82. doi: 10.1037/a0032671 PMC3844011 23607429

[31] Sicorello M Dieckmann L Moser D Lux V Luhmann M Schlotz W. Oxytocin and the stress buffering effect of social company: A genetic study in daily life. Soc Cogn Affect Neurosci. (2020) 15:293–301. doi: 10.1093/scan/nsaa034 32227088 PMC7235964

[32] de Jong TR Menon R Bludau A Grund T Biermeier V Klampfl SM. Salivary oxytocin concentrations in response to running, sexual self-stimulation, breastfeeding and the TSST: The Regensburg Oxytocin Challenge (ROC) study. Psychoneuroendocrinology. (2015) 62:381–8. doi: 10.1016/j.psyneuen.2015.08.027 26385109

[33] Alley J Diamond LM Lipschitz DL Grewen K. Associations between oxytocin and cortisol reactivity and recovery in response to psychological stress and sexual arousal. Psychoneuroendocrinology. (2019) 106:47–56. doi: 10.1016/J.PSYNEUEN.2019.03.031 30954918

[34] Tabak BA Leng G Szeto A Parker KJ Verbalis JG Ziegler TE. Advances in human oxytocin measurement: Challenges and proposed solutions. Mol Psychiatry. (2023) 28(1):127–140. doi: 10.1038/s41380-022-01719-z PMC9812775 35999276

[35] Brown CA Cardoso C Ellenbogen MA. A meta-analytic review of the correlation between peripheral oxytocin and cortisol concentrations. Front Neuroendocrinol. (2016) 43:19–27. doi: 10.1016/j.yfrne.2016.11.001 27836673

[36] Cardoso C Kingdon D Ellenbogen MA. A meta-analytic review of the impact of intranasal oxytocin administration on cortisol concentrations during laboratory tasks: Moderation by method and mental health. Psychoneuroendocrinology. (2014) 49:161–70. doi: 10.1016/j.psyneuen.2014.07.014 25086828

[37] Kelsch CB Ironson G Szeto A Kremer H Schneiderman N Mendez AJ. The relationship of spirituality, benefit finding, and other psychosocial variables to the hormone oxytocin in HIV/AIDS. In: Research in the Social Scientific Study of Religion, vol. 24. Brill, Leiden, Netherlands (2013). p. 137–62. doi: 10.1163/9789004252073_007

[38] Holbrook C Hahn-Holbrook J Holt-Lunstad J. Self-reported spirituality correlates with endogenous oxytocin. Psychol Religion Spirituality. (2015) 7:46–50. doi: 10.1037/a0038255

[39] Van Cappellen P Way BM Isgett SF Fredrickson BL. Effects of oxytocin administration on spirituality and emotional responses to meditation. Soc Cogn Affect Neurosci. (2016) 11:1579–87. doi: 10.1093/scan/nsw078 PMC5040919 27317929

[40] Bellosta-Batalla M Blanco-Gandía MC Rodríguez-Arias M Cebolla A Pérez-Blasco J Moya-Albiol L. Increased salivary oxytocin and empathy in students of clinical and health psychology after a mindfulness and compassion-based intervention. Mindfulness. (2020) 11:1006–17. doi: 10.1007/s12671-020-01316-7

[41] Bellosta-Batalla M Blanco-Gandía MC Rodríguez-Arias M Cebolla A Pérez-Blasco J Moya-Albiol L. Brief mindfulness session improves mood and increases salivary oxytocin in psychology students. Stress Health. (2020) 36:469–77. doi: 10.1002/smi.2942 32227624

[42] Hoehne K Vrtička P Engert V Singer T. Plasma oxytocin is modulated by mental training, but does not mediate its stress-buffering effect. Psychoneuroendocrinology. (2022) 141:105734. doi: 10.1016/j.psyneuen.2022.105734 35367715

[43] O’Halloran JP Jevning R Wilson AF Skowsky R Walsh RN Alexander C. Hormonal control in a state of decreased activation: Potentiation of arginine vasopressin secretion. Physiol Behav. (1985) 35:591–5. doi: 10.1016/0031-9384(85)90146-5 3906711

[44] Kabat-Zinn J. Full catastrophe living: Using the wisdom of your body and mind to face stress, pain, and illness. Bantam Books, New York, NY (2013).

[45] King BG Conklin QA Zanesco AP Saron CD. Residential meditation retreats: Their role in contemplative practice and significance for psychological research. Curr Opin Psychol. (2019) 28:238–44. doi: 10.1016/j.copsyc.2018.12.021 30878004

[46] Sahdra BK Ciarrochi J Parker PD Marshall S Heaven P. Empathy and nonattachment independently predict peer nominations of prosocial behavior of adolescents. Front Psychol. (2015) 6:263. doi: 10.3389/fpsyg.2015.00263 25852590 PMC4365438

[47] Wallace BA. The Attention Revolution. Wisdom Publications, Boston, Massachusetts (2006).

[48] Sahdra BK MacLean K Ferrer E Shaver PR Rosenberg EL Jacobs TL. Enhanced response inhibition during intensive meditation training predicts improvements in self-reported adaptive socioemotional functioning. Emotion (Washington D.C.). (2011) 11:299–312. doi: 10.1037/a0022764 21500899

[49] Zanesco AP King BG Powers C De Meo R Wineberg K MacLean KA. Modulation of event-related potentials of visual discrimination by meditation training and sustained attention. J Cogn Neurosci. (2019) 31:1184–204. doi: 10.1162/jocn_a_01419 31059348

[50] King BG Zanesco AP Skwara AC Saron CD. Cultivating concern for others: Meditation training and motivated engagement with human suffering. J Exp Psychology: Gen. (2023) 152:2897–924. doi: 10.1037/xge0001431 37166841

[51] Zanesco AP King BG Conklin QA Saron CD. The occurrence of psychologically profound, meaningful, and mystical experiences during a month-long meditation retreat. Mindfulness. (2023) 14(3):606–21. doi: 10.1007/s12671-023-02076-w

[52] Conklin QA King BG Zanesco AP Lin J Hamidi AB Pokorny JJ. Insight meditation and telomere biology: The effects of intensive retreat and the moderating role of personality. Brain Behavior Immun. (2018) 70:233–45. doi: 10.1016/j.bbi.2018.03.003 29518528

[53] Jacobs TL Epel ES Lin J Blackburn EH Wolkowitz OM Bridwell DA. Intensive meditation training, immune cell telomerase activity, and psychological mediators. Psychoneuroendocrinology. (2011) 36:664–81. doi: 10.1016/j.psyneuen.2010.09.010 21035949

[54] Álvarez-López MJ Conklin QA Cosín-Tomás M Shields GS King BG Zanesco AP. Changes in the expression of inflammatory and epigenetic-modulatory genes after an intensive meditation retreat. Compr Psychoneuroendocrinology. (2022) 11:100152. doi: 10.1016/j.cpnec.2022.100152 PMC9270205 35818436

[55] Conklin QA Patterson CE King BG Zanesco AP Pokorny JJ Álvarez-López MJ. Serum BDNF predicts increases in telomere length during a month-long residential meditation retreat. Brain Behav Immun Integr. (2023) 4:100023. doi: 10.1016/j.bbii.2023.100023

[56] Goldstein J Kornfield J. Seeking the Heart of Wisdom: The Path of Insight Meditation. Shambhala Publications, Boulder, CO (2001).

[57] Wallace BA. Boundless Heart: Cultivation of the Four Immeasurables. Snow Lion Publications, Ithaca, New York (1999).

[58] Feldman C. Boundless Heart: The Buddha’s Path of Kindness, Compassion, Joy, and Equanimity. Shambala Publications, Boulder, CO, Shambhala (2017). Available at: https://books.google.com/books?id=l7v1DQAAQBAJ.

[59] Leng G Sabatier N. Measuring oxytocin and vasopressin: bioassays, immunoassays and random numbers. J Neuroendocrinol. (2016) 28(10). doi: 10.1111/jne.12413 PMC5096068 27467712

[60] Szeto A McCabe PM Nation DA Tabak BA Rossetti MA McCullough ME. Evaluation of enzyme immunoassay and radioimmunoassay methods for the measurement of plasma oxytocin. Psychosomatic Med. (2011) 73:393–400. doi: 10.1097/PSY.0b013e31821df0c2 PMC3118424 21636661

[61] Brandtzaeg OK Johnsen E Roberg-Larsen H Seip KF MacLean EL Gesquiere LR. Proteomics tools reveal startlingly high amounts of oxytocin in plasma and serum. Sci Rep. (2016) 6:31693. doi: 10.1038/srep31693 27528413 PMC4985690

[62] Gnanadesikan GE Hammock EAD Tecot SR Carter CS MacLean EL. Specificity of plasma oxytocin immunoassays: A comparison of commercial assays and sample preparation techniques using oxytocin knockout and wildtype mice. Psychoneuroendocrinology. (2021) 132:105368. doi: 10.1016/j.psyneuen.2021.105368 34364024 PMC8487999

[63] Lafontaine M-F Brassard A Lussier Y Valois P Shaver PR Johnson SM. Selecting the Best Items for a Short-Form of the Experiences in Close Relationships Questionnaire. (2016) 32(2):140–54. doi: 10.1027/1015-5759/a000243.

[64] Spielberger C Gorsuch R Lushene R Vagg P Jacobs G. Manual for the State-Trait Anxiety Inventory (Form Y). Consulting Psychologists Press, Palo Alto, CA (1983).

[65] Goldberg LR. The structure of phenotypic personality traits. Am Psychol. (1993) 48(1):26–34. 10.1037//0003-066x.48.1.26 8427480

[66] Walker S Sechrist K Pender N. The health-promoting lifestyle profile: development and psychometric characteristics. Nurse Res. (1987) 36:76–81. 3644262

[67] Leys C Ley C Klein O Bernard P Licata L. Detecting outliers: Do not use standard deviation around the mean, use absolute deviation around the median. J Exp Soc Psychol. (2013) 49(4):764–6. doi: 10.1016/j.jesp.2013.03.013

[68] Benjamini Y Krieger AM Yekutieli D. Adaptive linear step-up procedures that control the false discovery rate. Biometrika. (2006) 93:491–507. doi: 10.1093/biomet/93.3.491

[69] Marsh N Marsh AA Lee MR Hurlemann R. Oxytocin and the neurobiology of prosocial behavior. Neuroscientist. (2021) 27:604–19. doi: 10.1177/1073858420960111 PMC8640275 32981445

[70] Bartz JA Zaki J Bolger N Ochsner KN. Social effects of oxytocin in humans: Context and person matter. Trends Cogn Sci. (2011) 15:301–9. doi: 10.1016/j.tics.2011.05.002 21696997

[71] Taylor SE. Tend and befriend: Biobehavioral bases of affiliation under stress. Curr Dir psychol Sci. (2006) 15:273–7. doi: 10.1111/j.1467-8721.2006.00451.x

[72] Neumann ID Slattery DA. Oxytocin in general anxiety and social fear: A translational approach. Biol Psychiatry. (2016) 79:213–21. doi: 10.1016/j.biopsych.2015.06.004 26208744

[73] Taylor SE. Tend and befriend theory. In: Handbook of theories of social psychology, vol. 1. Sage Publications Ltd, Thousand Oaks, California (2012). p. 32–49. doi: 10.4135/9781446249215.n3

[74] De Dreu CKW. Oxytocin modulates cooperation within and competition between groups: An integrative review and research agenda. Hormones Behav. (2012) 61:419–28. doi: 10.1016/j.yhbeh.2011.12.009 22227278

[75] De Dreu CKW Kret ME. Oxytocin conditions intergroup relations through upregulated in-group empathy, cooperation, conformity, and defense. Biol Psychiatry. (2016) 79:165–73. doi: 10.1016/j.biopsych.2015.03.020 25908497

[76] Shamay-tsoory SG Abu-akel A. The social salience hypothesis of oxytocin. Biol Psychiatry. (2016) 79:194–202. doi: 10.1016/j.biopsych.2015.07.020 26321019

[77] Harari-Dahan O Bernstein A. A general approach-avoidance hypothesis of Oxytocin: Accounting for social and non-social effects of oxytocin. Neurosci Biobehav Rev. (2014) 47:506–19. doi: 10.1016/j.neubiorev.2014.10.007 25454355

[78] Alaerts K Taillieu A Daniels N Soriano JR Prinsen J. Oxytocin enhances neural approach towards social and non-social stimuli of high personal relevance. Sci Rep. (2021) 11. doi: 10.1038/s41598-021-02914-8 PMC8655079 34880300

[79] Quintana DS Guastella AJ. An allostatic theory of oxytocin. Trends Cogn Sci. (2020) 24:515–28. doi: 10.1016/j.tics.2020.03.008 32360118

[80] Salzberg S. Mindfulness and loving-kindness. Contemp Buddhism. (2011) 12:177–82. doi: 10.1080/14639947.2011.564837

[81] Condon P. Meditation in context: Factors that facilitate prosocial behavior. Curr Opin Psychol. (2019) 28:15–9. doi: 10.1016/j.copsyc.2018.09.011 30359936

[82] Condon P Makransky J. Recovering the relational starting point of compassion training: A foundation for sustainable and inclusive care. Perspect psychol Sci. (2020) 15:1346–62. doi: 10.1177/1745691620922200 32745440

[83] Wilson-Mendenhall CD Dunne JD Davidson RJ. Visualizing compassion: episodic simulation as contemplative practice. Mindfulness. (2023) 14:2532–48. doi: 10.1007/s12671-022-01842-6 PMC10655951 37982041

[84] Zhang K Fan Y Yu R Tian Y Liu J Gong P. Intranasal oxytocin administration but not peripheral oxytocin regulates behaviors of attachment insecurity: A meta-analysis. Psychoneuroendocrinology. (2021) 132:105369. doi: 10.1016/j.psyneuen.2021.105369 34340132

[85] Fabian M Forsling ML Jones JJ Pryor JS. The clearance and antidiuretic potency of neurohypophysial hormones in man, and their plasma binding and stability. J Physiol. (1969) 204:653–68. doi: 10.1113/jphysiol.1969.sp008937 PMC1351579 5824107

[86] Vankrieken L Godart A Thomas K. Oxytocin determination by radioimmunoassay. Gynecologic Obstetric Invest. (1983) 16:180–5. doi: 10.1159/000299248 6618287

[87] Triki Z Daughters K De Dreu CKW. Oxytocin has ‘tend-and-defend’ functionality in group conflict across social vertebrates. Philos Trans R Soc B: Biol Sci. (2022) 377:20210137. doi: 10.1098/rstb.2021.0137 PMC8977669 35369742

[88] De Dreu CKW Greer LL Van Kleef GA Shalvi S Handgraaf MJJ. Oxytocin promotes human ethnocentrism. Proc Natl Acad Sci United States America. (2011) 108:1262–6. doi: 10.1073/pnas.1015316108 PMC3029708 21220339

[89] De Dreu CKW Greer LL Handgraaf MJJ Shalvi S Van Kleef GA Baas M. The neuropeptide oxytocin regulates parochial altruism in intergroup conflict among humans. Sci (New York N.Y.). (2010) 328:1408–11. doi: 10.1126/science.1189047 20538951

[90] Zhang H Gross J De Dreu C Ma Y. Oxytocin promotes coordinated out-group attack during intergroup conflict in humans. eLife. (2019) 8:e40698. doi: 10.7554/eLife.40698 30681410 PMC6347450

[91] Steinman MQ Duque-Wilckens N Trainor BC. Complementary Neural Circuits for Divergent Effects of Oxytocin: Social Approach Versus Social Anxiety. Biological Psychiatry (2019) 85(10):792–801. doi: 10.1016/j.biopsych.2018.10.008 PMC6709863 30503164

[92] Pagis M. Evoking Equanimity: Silent Interaction Rituals in Vipassana Meditation Retreats. (2015) 38:39–56. doi: 10.1007/s11133-014-9295-7.

[93] Holt-Lunstad J Birmingham W Light KC. Influence of a “warm touch” support enhancement intervention among married couples on ambulatory blood pressure, oxytocin, alpha amylase, and cortisol. Psychosomatic Med. (2008) 70:976–85. doi: 10.1097/PSY.0b013e318187aef7 18842740

[94] Seltzer LJ Ziegler TE Pollak SD. Social vocalizations can release oxytocin in humans. Proc Biol Sci / R Soc. (2010) 277:2661–6. doi: 10.1098/rspb.2010.0567 PMC2982050 20462908

[95] Ebitz RB Platt ML. An evolutionary perspective on the behavioral consequences of exogenous oxytocin application. Front Behav Neurosci. (2014) 7:225. doi: 10.3389/fnbeh.2013.00225 24478646 PMC3894461

[96] Kemp AH Guastella AJ. Oxytocin: prosocial behavior, social salience, or approach-related behavior? Biol Psychiatry. (2010) 67(6):e33–e34. doi: 10.1016/j.biopsych.2009.11.019 20060102

[97] Garland EL Atchley RM Hanley AW Zubieta J-K Froeliger B. Mindfulness-Oriented Recovery Enhancement remediates hedonic dysregulation in opioid users: Neural and affective evidence of target engagement. Sci Adv. (2019) 5:eaax1569. doi: 10.1126/sciadv.aax1569 31663023 PMC6795512

[98] Garland EL Fix ST Hudak JP Bernat EM Nakamura Y Hanley AW. Mindfulness-Oriented Recovery Enhancement remediates anhedonia in chronic opioid use by enhancing neurophysiological responses during savoring of natural rewards. psychol Med. (2023) 53:2085–94. doi: 10.1017/S0033291721003834 PMC10106294 37310337

[99] Lin S-H Chen PS Lee L-T Lee S-Y Tsai HC Chen WT. The association between the level of plasma oxytocin and craving among former heroin users. Eur Addict Res. (2018) 24:71–8. doi: 10.1159/000485563 29902803

[100] Quintana DS Rokicki J van der Meer D Alnæs D Kaufmann T Córdova-Palomera A. Oxytocin pathway gene networks in the human brain. Nat Commun. (2019) 10:668. doi: 10.1038/s41467-019-08503-8 30737392 PMC6368605

[101] Welch MG Margolis KG Li Z Gershon MD. Oxytocin regulates gastrointestinal motility, inflammation, macromolecular permeability, and mucosal maintenance in mice. Am J Physiology-Gastrointestinal Liver Physiol. (2014) 307:G848–62. doi: 10.1152/ajpgi.00176.2014 PMC4200316 25147234

[102] Chaves VE Tilelli CQ Brito NA Brito MN. Role of oxytocin in energy metabolism. Peptides. (2013) 45:9–14. doi: 10.1016/j.peptides.2013.04.010 23628372

[103] Li T Wang P Wang SC Wang Y-F. Approaches mediating oxytocin regulation of the immune system. Front Immunol. (2017) 7:693. doi: 10.3389/fimmu.2016.00693 28119696 PMC5223438

[104] Gutkowska J Jankowski M Antunes-Rodrigues J. The role of oxytocin in cardiovascular regulation. Braz J Med Biol Res = Rev Bras Pesquisas Medicas E Biologicas. (2014) 47:206–14. doi: 10.1590/1414-431X20133309 PMC3982941 24676493

[105] Watts HE. Seasonal regulation of behaviour: What role do hormone receptors play? Proc R Soc B: Biol Sci. (2020) 287:20200722. doi: 10.1098/rspb.2020.0722 PMC7423477 32635860

[106] Wikström Frisén L Larsson P Mincheva Nilsson L Henriksson Larsén K. Mood and Oxytocin Blood Levels in Physically Active Women with and without Oral Contraceptive Use in Relation to Seasonal Daylight Variation. (2017). Available online at: https://clinmedjournals.org/articles/ijsem/international-journal-of-sports-and-exercise-medicine-ijsem-3–058.php?jid=ijsem.

[107] Salonia A Nappi RE Pontillo M Daverio R Smeraldi A Briganti A. Menstrual cycle-related changes in plasma oxytocin are relevant to normal sexual function in healthy women. Hormones Behav. (2005) 47:164–9. 10.1016/j.yhbeh.2004.10.002 15664019

[108] Engel S Klusmann H Ditzen B Knaevelsrud C Schumacher S. Menstrual cycle-related fluctuations in oxytocin concentrations: A systematic review and meta-analysis. Front Neuroendocrinol. (2019) 52:144–55. doi: 10.1016/j.yfrne.2018.11.002 30458185

[109] Mccullough ME Churchland PS Mendez AJ Smith P Mendez AJ. Problems with measuring peripheral oxytocin: Can the data on oxytocin and human behavior be trusted? Neurosci Biobehav Rev. (2013) 37:1485–92. doi: 10.1016/j.neubiorev.2013.04.018 23665533

[110] Horvat-gordon M Granger DA Schwartz EB Nelson VJ Kivlighan KT. Oxytocin is not a valid biomarker when measured in saliva by immunoassay. Physiol Behav. (2005) 84(3):445–8. doi: 10.1016/j.physbeh.2005.01.007 15763582

[111] Lefevre A Mottolese R Dirheimer M Mottolese C Duhamel JR Sirigu A. A comparison of methods to measure central and peripheral oxytocin concentrations in human and non-human primates. Sci Rep. (2017) 7:1–10. doi: 10.1038/s41598-017-17674-7 29222505 PMC5722864

[112] Isgett SF Algoe SB Boulton AJ Way BM Fredrickson BL. Common variant in OXTR predicts growth in positive emotions from loving-kindness training. Psychoneuroendocrinology. (2016) 73:244–51. doi: 10.1016/j.psyneuen.2016.08.010 PMC5359600 27543885

[113] Hurlemann R Patin A Onur OA Cohen MX Baumgartner T Metzler S. Oxytocin enhances amygdala-dependent, socially reinforced learning and emotional empathy in humans. J Neurosci. (2010) 30:4999–5007. doi: 10.1523/JNEUROSCI.5538-09.2010 20371820 PMC6632777

